# Causal AI in Cardiac Arrhythmia: From Pattern Recognition to Mechanistic Insight

**DOI:** 10.1002/clc.70293

**Published:** 2026-04-07

**Authors:** Bara AbuBaha, Samia Aldwaik, Sarah Saife, Yousef Mahmoud‐Barqawi, Layan Omar, Omar Sawafta, Mohannad Sawalha, Hafez Nassar, Zeyad Alqasem, Karmel Khuffash, Hossam Salameh, Mohammed AbuBaha

**Affiliations:** ^1^ Department of Medicine An‐Najah National University Nablus Palestine

**Keywords:** arrhythmia, atrial fibrillation, causal AI, deep learning, explainable AI

## Abstract

**Introduction:**

Cardiac arrhythmias remain a leading cause of cardiovascular morbidity and mortality worldwide, and conventional diagnostic tools such as electrocardiography and Holter monitoring may fail to detect transient or asymptomatic events. Recent advances in artificial intelligence (AI) and machine learning have enhanced arrhythmia detection, risk stratification, and treatment planning; however, most existing models rely primarily on statistical associations rather than underlying physiological mechanisms.

**Methods:**

This narrative review was conducted through a structured but non‐systematic literature search of PubMed, Scopus, and Google Scholar, covering studies published between 2000 and 2025. Search terms included combinations of “cardiac arrhythmia,” “atrial fibrillation,” “causal inference,” “structural causal models,” “digital twins,” “mechanistic modeling,” “cardiac electrophysiology modeling,” and “artificial intelligence.” Peer‐reviewed articles were included if they demonstrated methodological depth and addressed causal inference or mechanistic modeling approaches in cardiovascular research, particularly in arrhythmia detection, risk prediction, treatment optimization, or clinical validation frameworks. Studies were prioritized based on methodological rigor, translational relevance, and recency. Editorials lacking methodological detail, non‐English publications, and studies relying solely on predictive models without incorporating causal or mechanistic components were excluded.

**Results:**

Causal artificial intelligence (Causal AI), offers a more mechanistically grounded framework for understanding arrhythmogenesis and therapeutic outcomes. Emerging evidence suggests that integrating clinical data with structural causal models, mechanistic modeling, and patient‐specific digital twins can bridge the gap between predictive performance and physiological interpretability. These approaches show promise in predicting ablation success, guiding therapy, and improving individualized care.

**Conclusion:**

Despite this potential, clinical implementation remains limited due to data heterogeneity, validation challenges, and regulatory constraints.

## Graphical abstract

Causal artificial intelligence (AI) in Cardiac Arrhythmia: From Pattern Recognition to Mechanistic Insight. Multimodal cardiac data, including electrocardiograms (ECG), wearable device recordings, cardiac imaging, genetic information, and electronic health records, are integrated to support AI‐based analysis. Traditional AI approaches primarily rely on pattern recognition and correlation‐based predictions. In contrast, causal AI models incorporate causal inference and structural causal modeling to identify cause effect relationships and generate mechanistic insights. These approaches can improve early arrhythmia detection, risk prediction, treatment planning, and patient‐specific cardiac modeling, ultimately contributing to more precise and personalized cardiac care. Key challenges include data heterogeneity, the need for rigorous clinical validation, and regulatory considerations.

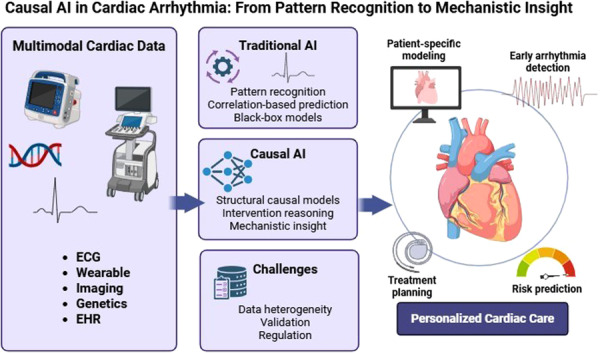

**Abbreviations**
AF: atrial fibrillation
AI: artificial intelligence
ECG: electrocardiogram
ML: machine learning
SCM: structural causal model

## 1 Introduction

Cardiac arrhythmias remain major contributors to global cardiovascular morbidity and mortality, acting as key drivers of stroke, heart failure, and sudden cardiac death, especially atrial fibrillation (AF) and ventricular tachyarrhythmias [1]. Traditional diagnostic tools, such as electrocardiogram (ECG) recordings and 24‐h Holter monitoring, frequently miss transient or asymptomatic arrhythmic episodes. Therefore, more sensitive and scalable diagnostic system is urgently needed to overcome this limitation [2]. Throughout the past few years, breakthroughs in AI and machine learning (ML) have revolutionized the way arrhythmia care is delivered, starting with early detection and risk assessment, all the way to optimizing treatment for each individual.

Analyzing an ECG using artificial intelligence (AI) can be as accurate as, and sometimes even better than, consulting with a cardiologist [3]. As a result of deep learning architectures, it is possible to extract hidden waveform characteristics that are beyond the ability of human perception and can even identify patients who may develop paroxysmal AF while still in sinus rhythm [4]. In addition, wearable devices equipped with AI—including a single‐lead ECG sensor or a photoplethysmography sensor have been reported to detect AF with sensitivity and specificity exceeding 90% [2, 5]. Study results, such as that of the Apple Heart Study, support the feasibility of AI‐based rhythm surveillance. However, practical issues such as false‐positive notifications and inadequate follow‐up remain [6].

With ML‐driven predictive analytics, AF progression and thromboembolic events can now be predicted by combining demographic, clinical, imaging, and genetic information [7, 8]. CHA2DS2‐VASc and other traditional scoring systems have been consistently outperformed by integrative models [7, 8]. In comparison to conventional methods, AI‐derived risk models have improved the accuracy of predicting post‐operative AF and stroke [8, 9].

The use of AI in therapeutic applications enables tailored treatment selection and procedural planning based on data‐driven decision‐making. At 12 months, patients who underwent ablation guided by AI‐detected atrial targets were significantly more likely to have no AF than those who underwent standard pulmonary vein isolation (88% vs. 70%, *p* 0.001) [10]. Similar to the Volta VX1, AI‐based electrogram interpretation systems enhance mapping accuracy and procedural safety [10]. In the clinical setting, AI is still a challenging technology to adopt. Most algorithms are developed based on small and demographically confined data sets, which makes them less generalizable [11]. The “black‐box problem” causes clinical experts to be hesitant about using deep learning models [11]. It is still a challenge for the path toward integration to comply with regulation, cover data, secure it, avoid algorithmic bias, and be ethically accountable [1, 5, 11].

Explainable AI (XAI) and Causal AI represent related but conceptually distinct paradigms. XAI methods such as SHAP and LIME aim to improve transparency by identifying which features influence model outputs; however, these approaches primarily reflect statistical associations learned by predictive models rather than validated interventional effects [12, 13, 14]. In contrast, Causal AI is grounded in structural causal models, counterfactual reasoning, and interventional inference, as formalized in causal inference theory [15, 16, 17]. By explicitly modeling how outcomes change under interventions, Causal AI moves beyond attribution toward mechanistic and clinically actionable reasoning. Throughout this manuscript, these terms are used deliberately and non‐interchangeably.

We want to diagnose and treat arrhythmias with greater precision and flexibility and this may become possible by using AI and ML. However, we should follow careful testing, clear understanding of how they work, and smooth integration into existing healthcare systems, to reach the true value of these technologies. When these things are met, cardiovascular medicine can fully benefit from their transformative power.

## 2 Current AI Landscape in Cardiac Arrhythmia

AI has revolutionized arrhythmia research by automatically detecting and classifying cardiac rhythms. In the early days of ML, features were handcrafted, and classical algorithms were used, like support vector machines and random forests, to achieve strong diagnostic performance on benchmark data sets like MIT‐BIH and PhysioNet. As deep learning emerged, convolutional neural networks were capable of learning from raw ECG signals from end‐to‐end, and Transformer‐based architectures were developed which captured inter‐lead and inter‐temporal dependencies. The majority of models are still correlation‐driven and lack mechanistic interpretability in spite of these developments, which restricts their applicability in clinical judgment and physiological comprehension.

### 2.1 ML in Arrhythmia Detection

Acharya et al. conducted a retrospective computational study using the MIT‐BIH Arrhythmia Database, demonstrating that support vector machine classifiers can differentiate between normal sinus rhythm and arrhythmic beats using handcrafted ECG features, including RR intervals, QRS morphology, and heart rate variability indices, with an accuracy of over 95%. A handcrafted feature was developed by converting physiological understandings into numerical inputs, such as the fact that an irregular RR interval may indicate AF, and a prolonged QRS duration may indicate ventricular conduction delay [18].

According to Chazal et al. logistic regression and random forest algorithms trained on interval‐ and morphology‐based features were used to classify ventricular ectopic beats, reporting sensitivities exceeding 90%. It is noted that their approach emphasizes domain‐specific feature engineering, so the study demonstrated that classical ML methods can be used to detect arrhythmias when they are supported by expertise in cardiac electrophysiology [19].

In spite of this, these early approaches were constrained by certain limitations. As a result of manual design and tuning of features for each arrhythmia type, scalability between data sets and patient populations was limited. Therefore, while traditional ML achieved high accuracy when applied to standardized data sets like MIT‐BIH and PhysioNet, its performance often declined when applied to real‐world or heterogeneous data.

### 2.2 Deep Learning and End‐to‐End Pattern Recognition

Hannun et al. in a prospective validation study using over 90 000 single‐lead ECG recordings taken with wearable sensors, a convolutional neural network could accurately classify 12 rhythms with cardiologist‐level accuracy, with an average F1‐score of 0.83. The model learns directly from raw ECGs, demonstrating for the first time that deep learning can detect arrhythmias at expert levels without requiring handcrafted features [20].

According to Yildirim et al. the MIT‐BIH Arrhythmia Database was used to develop a hybrid convolutional‐long short‐term memory (CNN–LSTM) architecture that captured spatial morphology and temporal dependencies in ECG signal analysis. Sequential modeling was demonstrated to improve the detection of intermittent rhythm disorders such as AF and premature contractions by achieving 99% accurate results across several arrhythmia classes [21].

Dong et al. in a methodological development study, presented a Transformer‐based attention network for multi‐lead ECG classification. In addition to improving AF detection sensitivity by 6% over recurrence Neural Network (RNN), the model produced interpretable attention maps that highlight diagnostically relevant waveform regions, providing a step towards more transparent, clinically meaningful deep models utilizing self‐attention to capture long‐range and interlead dependencies [22].

All of these studies point out the shift from feature‐based to end‐to‐end deep learning, which can extract features from diverse ECG data, including multi‐lead recordings, Holter recordings, and wearable recordings automatically. However, mechanistic interpretation and clinical integration remain challenges.

### 2.3 Predictive Success Versus Mechanistic Understanding

Although deep learning models achieve high predictive accuracy, their decision‐making processes often remain opaque. Studies have shown that saliency and attribution methods can highlight ECG segments influencing model predictions; however, these regions do not always correspond to clinically meaningful electrophysiologic features [22]. Additionally, models may exploit spurious correlations, such as baseline artifacts, leading to misclassification of rhythms [23]. Even highly accurate systems capable of detecting AF from sinus rhythm ECGs may rely on surrogate statistical markers rather than causal mechanisms [24].

Together, these findings underscore the persistent gap between predictive performance and mechanistic understanding, reinforcing the need for approaches that integrate physiological modeling with causal reasoning.

### 2.4 Current Limitations of AI in Cardiology

Class imbalance, particularly the underrepresentation of rare arrhythmias, reduces model sensitivity when applied to heterogeneous clinical data sets [25]. As a result, less frequent but clinically significant rhythms may be misclassified. Addressing this requires strategies such as data augmentation, synthetic data generation, and class‐weighted optimization to improve robustness across arrhythmia categories.

Beyond performance concerns, regulatory requirements increasingly demand transparency and auditability. Frameworks such as the EU AI Act emphasize explainability for high‐risk healthcare applications, reinforcing that clinical adoption depends not only on accuracy but also on interpretability and accountability [26].

## 3 Foundations of Causal Artificial Intelligence (Causal AI)

**Figure 1:**
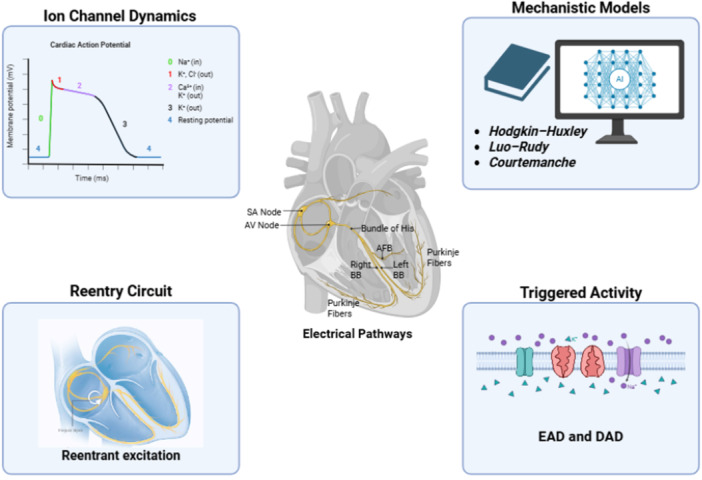
Mechanistic modeling links ionic currents, cellular dynamics, and tissue‐level conduction to arrhythmia generation mechanisms including reentry, triggered activity, and conduction block.

Causal AI is a sophisticated system that helps comprehend and represent the actual causes leading to the effects instead of just the associations that have been observed in the data. It sets up definite means for telling the difference between mere observation and an actual intervention, thus being the scientific ground for the application of data‐driven discoveries (Figure 1).

### 3.1 Defining Causality in Data Science

According to Pearl et al.'s the central conceptual leap for reasoning about interventions is the formal distinction between observing a variable and actively setting it, a distinction compactly expressed by the do(·) operator and its attendant interventional calculus. Consequently, the do‐calculus provides algebraic rules that permit certain interventional queries to be transformed into expressions, provided the causal graph correctly encodes the data‐generating assumptions and the required conditional distributions are estimable from the observed data, thus changing the informal “what‐if” questions into formal estimands [17]. However, Hernán‐Robins et al. have argued that the use of potential outcomes offers the most natural way to present clinical causal questions, because causal claims ultimately rest on contrasts between factual and counterfactual outcomes that researchers must identify [15]. Building on that emphasis, Imbens‐Rubin et al. pointed out that the potential‐outcomes framework is a “forcing” framework (in the sense that it necessitates the explicit declaration of the estimand, for example, average or conditional treatment effects) and makes clear the assumptions that are needed for identification from data (exchangeability, consistency, and positivity), thus connecting study design and analysis to the causal conclusion's validity [16].

### 3.2 Core Methods in Causal Inference

Pearl et al.'s study showed that directed acyclic graphs (DAGs) and structural causal models (SCMs) make causal assumptions in a very explicit way by depicting nodes, directed edges, and structural equations; accordingly, these pictorial representations directly guide identification strategies (e.g., the use of back‐door and front‐door criteria) by revealing confounding and mediation pathways [17]. In line with this practical value, Greenland et al. demonstrated that causal diagrams constitute a feasible toolkit for epidemiologic practice by creating minimal adjustment sets and revealing how classical concepts of confounding correspond to formal graphical criteria. Extending the scope of these tools, Peters et al. argued that contemporary causal formalisms reconnect scientific theories with algorithmic learning, thus offering principled ways of calculating interventional distributions as well as including structural priors in learning algorithms [27]. Nevertheless, VanderWeele et al. emphasized that when the aim is to elucidate mechanisms, mediation and effect decomposition must be framed in counterfactual terms; therefore, legitimate mediation analyses in observational studies require explicitly stated assumptions and sensitivity analyses to quantify how unmeasured confounding might bias estimates [28].

### 3.3 Causal Discovery From Observational Data

Spirtes et al. developed constraint‐based discovery algorithms (e.g., the PC family) and described how conditional‐independence patterns in observational data can be utilized to infer candidate causal skeletons for hypothesis generation; at the same time, it was noted that there's limits imposed by latent confounders and the faithfulness assumption [29]. Complementing constraint‐based methods, Shimizu et al. introduced LiNGAM and showed that, under linearity, absence of hidden confounders and non‐Gaussian disturbances, causal directions can be recovered where covariance‐based methods fail [30]. By contrast, Granger et al.'s lagged‐predictability criterion (Granger causality) and subsequent methodological work clarified that Granger tests examined directed predictive precedence in time series but do not, by themselves, establish mechanistic, intervention‐level causation [31]. More recently, Runge et al. extended causal discovery to nonlinear, nonstationary and high‐dimensional time series and they showed algorithms that significantly enhance detection power in realistic dynamical data sets, improvements that are quite relevant to physiological recordings such as multi‐lead ECG and continuous wearable signals [32]. Due to the fact that discovery algorithms are based on strong assumptions often violated in biological contexts, contemporary practice favors hybrid workflows that combine data‐driven discovery with mechanistic priors or simulator‐based validation to improve identifiability and physiological interpretability [27, 32].

### 3.4 Relationship Between XAI and Causal AI

Rudin et al. have cautioned that relying solely on post‐hoc explanations of black‐box models in high‐stakes settings is risky; consequently, when clinical actions follow from model outputs, inherently interpretable or causally constrained models are preferable [12]. To make attribution clearer, Lundberg‐Lee et al.'s proposed SHAP as a cohesive, axiomatic framework for local feature attribution that helps understand the idea of the attribution methods by addressing their strengths and weaknesses through the clarification of the attribution of single predictions with Shapley‐value decompositions [13]. Similarly, Ribeiro et al. came up with the idea of LIME and demonstrated that locally faithful surrogate models can make a single prediction understandable to users. Although it was pointed out that locality and stability limitations can be mistakenly interpreted as intervention effects when explanations are used as their proxies [14]. Taken together, methodological reviews have pointed out that the most widely used attribution methods frequently reflect associations learned by predictive models rather than validated interventional effects; therefore, such attributions should be used with caution and not relied on directly for clinical decisions [13, 14]. Accordingly, Pearl et al. along with other causal theorists, have claimed that it is necessary to incorporate SCMs, explicit counterfactual estimands, or interventional constraints into the model framework in order to make descriptive explanations convertible into actionable “what‐if” answers which clinicians can trust, audit, and use for decision‐making [17, 27] (Table 1).

**Table 1:** Comparative overview of key causal inference frameworks, outlining their theoretical foundations, strengths, limitations, and relevance to electrophysiology research and intervention modeling.

Method	Core mechanism	Advantages	Limitations	Key references
Directed acyclic graphs (DAGs)	Graph‐based representation of causal relations between electrophysiologic variables	Visual clarity of confounding and mediation; intuitive for clinical interpretation	Requires prior knowledge; may not capture latent confounders	[17]
Structural causal models (SCMs)	Encodes causal equations linking variables	Enables interventional reasoning and simulation of “what‐if” scenarios	Computationally intensive; dependent on model correctness	[17, 27]
Counterfactual/potential outcome framework	Compares observed vs hypothetical outcomes	Quantifies treatment effects (e.g., ablation success vs. control)	Needs strong assumptions (exchangeability, positivity)	[15, 16, 28]
Constraint‐based algorithms (e.g., PC, FCI)	Learns causal structure from statistical independencies	Suitable for hypothesis generation from ECG data sets	Limited by faithfulness assumption, noise sensitivity	[29, 32]
LiNGAM	Uses non‐Gaussianity to infer causal directionality	Handles linear data with identifiable direction	Assumes no hidden confounding	[30]

## 4 Mechanistic Insights in Cardiac Electrophysiology (Figure 2)

**Figure 2:**
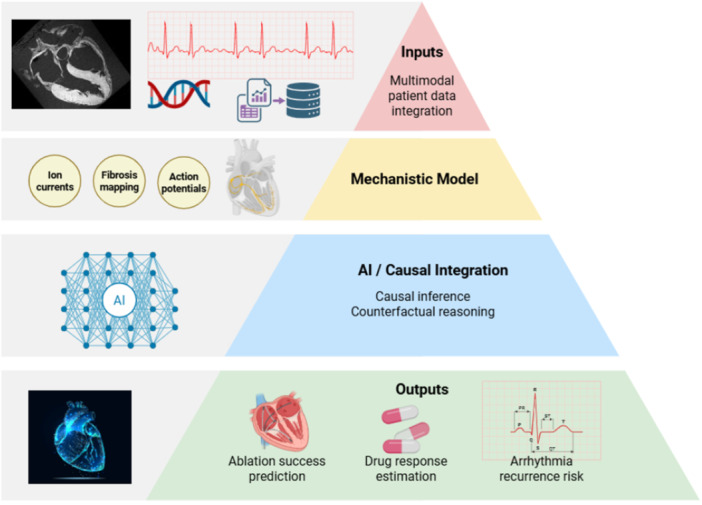
Hybrid mechanistic–AI integration combining physiological simulations with causal inference enables patient‐specific digital twins for arrhythmia prediction and therapy optimization.

Cardiac electrophysiology describes the mechanism behind the heart's electrical system, which generates and transmits impulses that control and coordinate cardiac contractions. Each cardiac cell maintains a negative resting membrane potential through potassium ion channels and the sodium‐potassium pump. When stimulated, voltage‐gated sodium channels open rapidly, allowing sodium ions to rush into the cell and depolarize it, thus starting the cardiac action potential or phase zero. This is then followed by calcium entry through L‐type calcium channels, which sustains a plateau phase and triggers calcium release from the sarcoplasmic reticulum, thus turning electrical activity into mechanical muscle contraction. Repolarization then occurs when potassium ions flow out of the cell and restore the resting potential, and this causes the heart to relax [33, 34, 35]. The electrical signals are organized through the cardiac conduction system. The process begins in the sinoatrial node in the right atrium, then travels to the atrioventricular node. The atrioventricular node causes a small delay due to fewer gap junctions and a smaller cell size, thus allowing the ventricles to fill with blood before contraction, and then it goes through the bundle of His and Purkinje fibers, which spread the electrical signals to both ventricles, causing a strong contraction which will pump blood through both the pulmonary and systemic circulations [36]. Any disruptions in this process will result in abnormal electrical impulses, thus resulting in arrhythmias.

The main mechanisms underlying arrhythmias include reentry, triggered activity, and conduction block. In reentry, electrical impulses reactivate an area of the heart that has already recovered from depolarization. This occurs due to the presence of scar tissue or fibrosis, such as in individuals with a history of myocardial infarction. The reentry of the signal results in abnormal activation of tissue and causes atrial flutter or ventricular tachycardia to occur. In triggered activity, abnormal activity occurs either during repolarization or immediately after. The first type of early afterdepolarizations (EADs) is usually caused by prolonged action potential, thus resulting in extra or premature beats and is linked to conditions with prolonged QT intervals, including torsades de pointes. The other type of triggered activity, delayed afterdepolarizations (DADs), is typically caused by calcium overload in the cell, resulting in the accumulation of calcium in the sarcoplasmic reticulum and spontaneous current to occur, causing depolarization. DADs are often seen in conditions such as heart failure or digitalis toxicity, where problems with calcium handling are seen. Finally, there are conduction blocks which result from structural abnormalities such as scarring or an ischemic injury, or functional abnormalities due to electrolyte imbalances or ion channel dysfunction. In a structural conduction block, electrical impulses travel around damaged areas, resulting in slower or unidirectional conduction, and this can feed into reentry circuits occurring. Both conduction blocks can promote reentrant excitation, and understanding the mechanisms behind arrhythmias allows for the development of biophysical models that can predict how small changes can affect cardiac rhythm [33, 37, 38, 39].

Mechanistic modeling has been important in understanding more about how cardiac behavior is affected by ionic and structural mechanisms. Early models, such as the Hodgkin‐Huxley framework from 1952, created a foundation that demonstrated how ion channels generate cardiac action potential. Then the Luo‐Rudy model was built on the Hodgkin‐Huxley equations and integrated information regarding potassium currents and sodium‐calcium dynamics. The Courtemanche et al. model refined this, thus creating a base for us to study atrial electrophysiology and arrhythmia mechanisms [35, 40, 41]. With time, these models evolved more into multiscale models that link cellular changes and translate them to the tissue and organ level.

Multiscale models bridge the gap between ion channel kinetics and cardiac behavior, making it possible to create simulations and explore mechanisms such as reentry or conduction block. Tusscher‐Panfilov and O'Hara–Rudy frameworks incorporated ventricular heterogeneity and human‐specific electrophysiological data [42]. Grandi et al. developed a model that emphasized atrial remodeling [43]. In Trayanova et al.'s work, it is mentioned how patient‐specific multiscale simulations, which integrate clinical imaging and electrophysiological data, result in better models [35]. Concerns regarding the validation and trustworthiness of models were raised by Pathmanathan and Gray, who noted that variations in tissue parameters and numerical methods can affect simulation outcomes, which is why we need standardized validation frameworks to ensure the validity and reliability of these models [44].

With the need for more complex mechanistic models, data‐driven approaches, and the integration of AI has been tried. The use of AI, which implements pattern recognition, can extract electrophysiological parameters from large‐scale data to reflect more patient‐specific cardiac behavior. Hybrid mechanistic‐AI frameworks can be used to reproduce cardiac activity and infer and understand relationships between cellular physiology and arrhythmic outcomes, which might have been overlooked if done so manually. AI can also be implemented to create personalized cardiac models for patients using their data, and thus, a digital twin of the heart can be used to predict arrhythmic risk and guide treatment [45, 46, 47]. This implementation will allow doctors to visualize possible outcomes before trying different therapeutic strategies on their patients.

Mechanistic modeling allows researchers to manipulate and observe certain changes that are difficult to isolate experimentally. These models offer precise control over ion channel behavior, making it possible to see causative relationships and arrhythmia mechanisms. By altering specific parameters such as gap‐junction coupling or calcium handling, researchers are able to see how each factor can affect cardiac rhythms, without the effect of external factors and confounding variables we see in real experiments. These biophysical simulations, however, should also be validated against experimental and clinical data to ensure reliability. The difference between the virtual lab results and the results of clinical data could be due to variability in models, but it could also provide additional understanding of how spatial resolution or parameter selection has affected the validity of this model.

Creating standardized frameworks for validation is important to ensure that these computerized models will be able to represent biological mechanisms and results, and not computational artifacts. Different studies have tested the credibility of different models. For example. Galappaththige et al. applied the ASME V&V40 framework and compared visual cohorts of ventricular simulations to real clinical recordings to see whether the model was able to predict similar to the observed outcomes [48]. Their results demonstrated a R‐squared value of 0.82–0.91 correlation between simulated and recorded values. Potse et al. also compared patient‐specific ventricular activation models to real recordings from patients with a left bundle branch block (LBBB) and demonstrated how altered electrical conduction can explain the LBBB patterns seen in these patients [49].

Mechanistic modeling is very important in understanding the mechanisms and relationships between small changes and cardiac behavior, and the integration of AI will enhance the current models by personalizing them and catching patterns that might have been missed. As these models advance, the gap between the theoretical knowledge we have about electrophysiology and clinical cardiology will be less, and more accurate predictions of arrhythmic risk and treatment plans will be applied.

## 5 Applications of Causal AI in Cardiac Arrhythmia (Figure 3)

**Figure 3:**
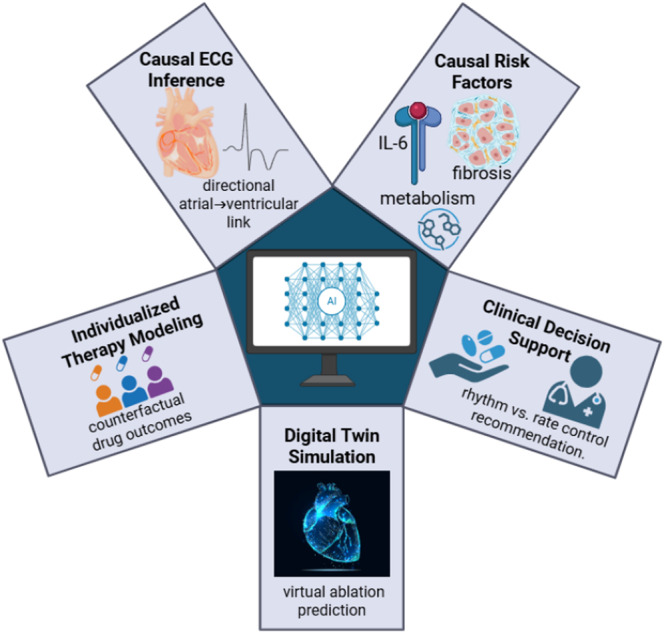
Causal AI applications span mechanistic risk factor identification, causal ECG analysis, individualized treatment modeling, digital twin simulations, and explainable clinical decision support.

Causal AI helps us not only to detect patterns but also to understand how exactly it happens that arrhythmias appear, get worse, or respond to treatment. Several practical examples can be observed in the current research: identifying the risk factors causally linked to a condition, finding relationships between the mechanisms of cardiac electrophysiology, predicting personal treatment responses, digital twin‐based simulation modeling, and creating decision‐support systems in clinical practice. While these methods are at different points in their development, they showcase the potential of causal reasoning to convert physiological knowledge into clinical actions.

Using causal inference in arrhythmia research can lead to the identification of risk factors at the biochemical or organ level that have a causal impact on disease development. Marcus et al. found that the interleukin‐6 signaling pathway promotes arrhythmias even in the absence of other risk factors through a direct pro‐arrhythmogenic effect using causal mediation analysis. This points to a possible mechanistic role of inflammatory pathways in AF [50]. Structural remodeling is likely another causative factor in arrhythmogenesis. Based on computational models generated from late‐gadolinium‐enhanced MRI, McDowell et al. illustrated that atrial fibrosis highly increases the risk of AF recurrence after ablation, independent of demographic factors like age or hypertension [51]. Metabolic disturbances are considered by experimental studies as a factor leading to electrophysiological remodeling. Animal experiments by Albarado‐Ibañez et al. confirmed that metabolic syndrome factors, specifically obesity and insulin resistance, could modify the sinoatrial node's electrical activity and thus be a source of arrhythmias [52]. In sum, these studies point towards the use of causal AI methods to dissect the interplay between inflammatory, structural, and metabolic pathways in the context of arrhythmia susceptibility.

Causal modeling has been one of the methods used for electrophysiological data, and it has helped to expose the directionality of cardiac electrical events. Using continuous electrocardiographic records, Zhao et al. designed computational methods for inferring causal dependencies between atrial depolarization instability and subsequent ventricular conduction delays, thus making it possible to predict paroxysmal AF episodes earlier [53]. In a similar vein, Pérez‐Riera et al. introduced a multimodal causal pattern that starts with ion‐channel polymorphisms and culminates in structural atrial remodeling and the subsequent alterations in P‐wave dispersion. This pattern serves as a pictorial representation of how genetic and electrophysiological influences can combine to foster arrhythmogenicity [54]. More experimental works give their nod to these mechanistic connections; De Col et al. for example, found that changes in the expression levels of sodium‐channel genes come before the detectable alterations in conduction velocity, thereby indicating that changes at the molecular level could be the primary causal factors of electrical conduction abnormalities [55]. All these discoveries serve as proof that causal reasoning techniques can be used to meld molecular, electrophysiological, and clinical data to build more complete mechanistic models of arrhythmia initiation.

Besides risk identification and mechanistic discovery, causal inference can be an important tool in the individual patient's treatment planning. Counterfactual modeling techniques empower scientists to predict how different therapeutic interventions could affect patient outcomes, given different scenarios. Pang et al. took advantage of targeted maximum likelihood estimation to analyze treatment effects and discovered that patients with prolonged PR intervals might not respond well to some class III antiarrhythmic drugs [56]. Electrophysiological investigations also hint that the efficacy of treatments can be conditioned on cardiac substrate features. Burashnikov et al. found out that amiodarone burns down the possibility of AF recurrence in patients exhibiting a high degree of atrial conduction heterogeneity, regardless of patient age or other health conditions [57]. In the same way, digital modeling strategies that take into account substrate‐level parameters have been employed for predicting ablation outcomes in various patient cohorts, thus displaying the powerful side of causal AI in the realm of personalized medicine [58]. It is true that quite a few of these tools are still in the phase of experimentation, but they serve as an excellent showcase of the role that causal inference can play in precision electrophysiology 1 day.

Simulation‐based modeling is another area where causal AI can be effectively employed. Digital twin technology enables researches to create virtual patient‐specific heart models that reflect electrophysiological behavior under different intervention scenarios. Sakata et al. showed that digital heart twins implementing causal inference are able to mimic electrophysiological interventions like targeted ablation of reentrant circuits, thus predicting arrhythmia termination in the living organism with great accuracy [59]. In fact, Chiu et al., in a related study, employed neural‐network techniques that integrate causal graphs with physics‐informed models so as to determine the counterfactual effects of altering ion‐channel conductance in simulated electrophysiological settings [60]. Besides, these simulation environments allow not only the testing of causal assumptions in a controlled setting but also the pre‐clinical evaluation of therapeutic strategies.

On the other hand, causal reasoning is also making its way into the building of clinical decision‐support systems for arrhythmia management. Ma et al. illustrated that the addition of causal relations to AF detection systems enables the identification of patient subgroups that are most likely to benefit from particular management strategies [61]. In the same way, Liang et al. found that causal decision networks, which are used in AF management, could demonstrate that patients with a smaller extent of structural remodeling derive more benefits from rhythm‐control strategies than the conventional rate‐control approaches [62]. The upshot of advances in device‐based therapies is the potential to perform causal decision‐making in real‐time. Li et al. shared their experience with implantable pacing systems, which are equipped with causal modeling units allowing the pacing parameters to be changed dynamically in response to the predicted counterfactual outcomes. This, in turn, led to more effective arrhythmia suppression without a rise in adverse effects [63].

These uses show the big promise of causal AI for arrhythmia care at the different stages, from identifying mechanisms to finding the best treatment, but only a few of the implementations are still in computer or early experimental stages. Validation in clinical trials is still minimal, and most of the systems need a lot of work before they can be used as usual in the electrophysiology clinic. However, the sum total of these studies points to causal AI as a connecting link between detailed cardiovascular mechanism studies and efficient clinical decision‐making by converting complicated electrophysiological data to understandable, patient‐specific observations (Table 2).

**Table 2:** Summary of current applications of Causal AI in cardiac arrhythmia, detailing core mechanisms, clinical benefits, and translational constraints.

Application area	Core mechanism	Key findings/benefits	Limitations	References
Causal risk factor identification	Mediation and causal path modeling	Identified IL‐6, fibrosis, and metabolic pathways as direct arrhythmic causes	Observational bias and confounding	[54, 55, 56]
ECG‐based causal discovery	Temporal and directional dependency modeling	Early AF prediction and linking ion‐channel polymorphisms to ECG features	Limited interpretability and dataset diversity	[57, 58, 59]
Individualized treatment response	Counterfactual models and target maximum likelihood estimation	Estimated subgroup‐specific drug response and ablation outcomes	Data sparsity, need for interventional validation	[60, 61, 62]
Digital heart twins	Patient‐specific mechanistic + causal simulations	Virtual ablation predicted clinical success	High computational cost	[63, 64, 65]
Clinical decision support	Causal decision networks integrated into EHR	Improved rhythm‐control selection and device optimization	Regulatory and interpretability hurdles	[66, 67, 68]

## 6 Challenges and Limitations (Figure 4)

**Figure 4:**
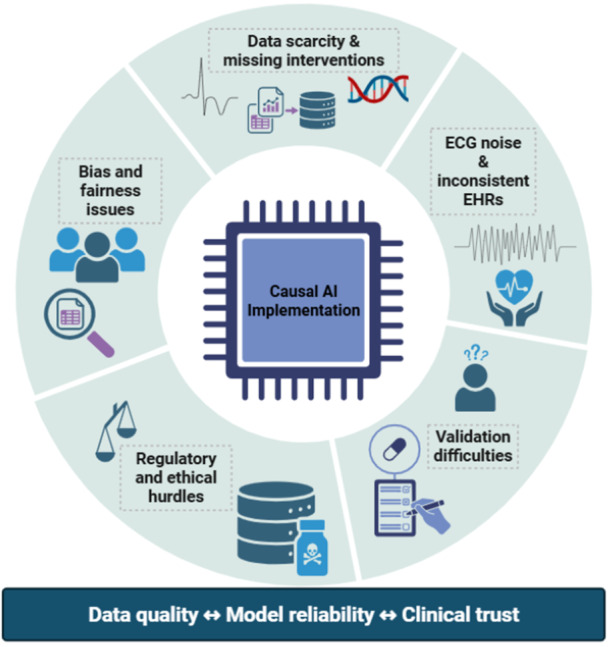
Challenges limiting causal AI in electrophysiology include incomplete data, noise, lack of validation, regulatory barriers, and fairness concerns that affect model reliability and clinical trust.

Many researchers have turned their attention towards causal AI in arrhythmia management. However, a number of structural limitations continue to prevent its reliable clinical use. In fact, four major challenges keep coming up throughout the literature: the lack of good‐quality data, methodological and computational issues, the inability to validate causal claims properly, and ethical or regulatory concerns related to clinical deployment. Although lots of studies emphasize effective brainstorming solutions, everyone agrees that the present cardiovascular data and healthcare systems are still far from enabling powerful causal modeling.

### 6.1 Data‐Related Challenges

The great majority of the literature identifies one common thread: causal AI calls for a paradigm shift in the kinds of data sets that it requires as compared to standard predictive ML. In essence, predictive models are capable of functioning by extracting associations from past data, whereas causal models require experimental data that portray the changing of conditions or physiology leading to different results well (how a change in treatment or physiology leads to a change in outcome is the essence of causal data).

In fact, very few data are available in cardiovascular medicine sufficient for causal modeling, as several papers pinpoint. For example, Dhir et al. found that meta‐learning methods could help figure out the causal effects of interventions even if causal structures are not clear, but they also show that getting trustworthy results is a challenge when interventions are wrongly labeled or only partly documented [64]. This issue is even more acute in the field of arrhythmia management, where a lot of clinical decisions, for example, pacing adjustments, medication changes, and ablation strategies, are quite commonly recorded differently across various institutions. Similar issues are faced when heterogeneous intervention data sets are being brought together. For instance, Wang et al. have pointed out that the merging of several data sets that have different experimental targets or diverse definitions for interventions may trigger mixed causal structures as well as structural contradictions leading to model training failure [65]. Such inconsistencies make it difficult to develop universal causal models that would work across various institutions or device platforms.

Besides, the segmentation of temporal data represents an additional problem. To perform a longitudinal study on arrhythmia development, one needs complete patient histories, while documentation in a hospital is often episodic and incomplete at best. According to Coupland et al., fragmented electronic health records (EHRs) hamper our ability to trace the course of diseases throughout the life span, especially when important physiological events are not recorded as part of regular practice [66]. Implanted cardiac devices make an ongoing telemetry available, which, in principle, could be utilized to overcome such a deficiency. Nevertheless, contextual clinical data like the rationale behind the changes in therapies, for instance, are usually missing, which makes the device‐generated data less meaningful [67]. More generally, examinations of AI in cardiovascular medicine indicate that the majority of the current arrhythmia prediction models are based on retrospective data sets, which do not contain the level of detail relevant to interventions enough to support causal reasoning [68, 69]. Consequently, the vast majority of the systems at present still work on the basis of correlation‐supported prediction rather than causal understanding.

### 6.2 Methodological and Computational Barriers

Beyond data availability, methodological challenges also limit the performance and reliability of causal AI systems. One major issue is the heterogeneity and noise inherent in real‐world clinical data sets.

Electrocardiographic signals that come from wearable or ambulatory devices are likely to be contaminated with heavy noise from various sources, such as motion artifacts, muscle activity, or device‐related interference. Kalpande et al. show that even sophisticated noise‐detection algorithms find it difficult to generalize across data sets obtained in different conditions, thus underscoring the challenge of keeping model performance robust in real‐world environments [70]. Besides, electronic health records' structural variability adds more layers of complexity. To name a few, Chung et al. point out variables being inconsistently coded, non‐availability of some clinical variables, and patient histories being scattered are just a few reasons limiting the number of predictors AI models can have, and thus their ability to explain complex physiological interactions is also limited [71]. These problems are most serious when dealing with causal modeling that relies on the accurate representation of the relationships between multiple interacting variables.

Various research articles point to the fact that modeling robustness to this kind of variability can be enhanced by incorporating causal structure in the model design itself. For instance, Wang et al. show that the use of causal inference methods integrated into neural network structures can diminish the impact of noise and confounding elements in ECG classification tasks [72]. On the other hand, such a method is dependent on the availability of well‐annotated training data and the existence of definite causal assumptions. There are also some authors who stress the role of data preprocessing and standardization of data acquisition techniques. Rahman et al. explain that data augmentation methods cannot only deal with the problem of scarce training data but also help in generalizing the model better to different patient populations [73]. Besides, the overall review of AI in cardiac diagnostics also reflects that inconsistent data collection protocols from different institutions are one of the main hurdles that prevent reliable model deployment [74].

There is also a rising agreement on the fact that cardiovascular AI research needs to have stricter adherence to methodological standards. Van Royen et al. put forward a few quality norms for predictive models, like open and clear reports of the models, thorough and strict validation procedures, and development pipelines that can be reproduced [75]. These rules especially become very crucial when it comes to causal AI, where a wrong understanding of the cause‐effect relationship can mislead the whole system.

### 6.3 Validation and Interpretability

Another frequently mentioned limitation is the challenge of validating causal AI models. Traditional predictive systems, which are often evaluated by metrics like accuracy or area under the curve, don't quite match causal models since the latter need to prove that their inferred relationships correspond to actual physiological mechanisms. A number of papers have suggested simulation‐based frameworks as one of the validation strategies. For example, Sufian et al. have laid out the simulation pipelines for creating synthetic patient trajectories that can be used to test causal hypotheses in a controlled manner [76]. In fact, multi‐omics simulation environments have also been suggested for the purpose of evaluating AI‐generated biological pathways in cardiovascular disease, and these can serve as biologically motivated testbeds for algorithmic predictions [77].

Nonetheless, the debate is still open about whether interpretability methods are enough on their own to validate causal models. Like Salih et al., many believe XAI tools can help understand how models arrive at predictions, but that does not guarantee that the causal relationships they rest on are accurate [78]. If experimental or prospective validation is absent, explanations might be no more than statistical artifacts in the training data instead of genuine physiological mechanisms. So, the difference between interpretability and causality is still a very significant challenge from a methodological standpoint. AI systems typically offer feature importance scores or visual explanations, but these do not come with a guarantee that the relationships identified actually represent causal processes in cardiac electrophysiology.

### 6.4 Ethical, Regulatory, and Clinical Concerns

Besides technical issues, the use of causal AI in healthcare brings up a lot of ethical and legal dilemmas as well. Transparency and accountability are the major ethical issues that come up again and again, especially when it comes to making clinical decisions through AI.

Marey et al. point out that it is clinician trust and patient confidence that get jeopardized by black‐box models. This becomes more of an issue when AI outputs are used for treatment decisions in high‐risk cardiovascular conditions [79]. This concern is further raised by an electrophysiology factor where therapeutic decisions could be invasive or a device implantation. Bias and fairness problems still remain as critical issues. Investigations at large of AI systems in electrophysiology demonstrate that models trained on limited data sets might perform poorly in underrepresented populations, which include differences in sex, ethnicity, and device type [80]. Such disparities raise worries about equitable access to AI‐driven care and point out the necessity of diverse training data sets.

Several authors point out that AI should be a tool to support decision‐making in clinical settings rather than being a decision‐maker on its own. Mooghali et al. believe that clinicians have to keep the final say in treatment decisions. However, at the same time, they should be sufficiently aided to understand and critically evaluate AI outputs [81]. Legal experts also highlight that the present liability system is not capable of handling the distributed responsibility among developers, clinicians, and healthcare institutions when AI systems are used in making clinical decisions [82]. More generally, ethical use of AI calls for protections against algorithmic bias, misinterpretation, and over‐dependence on automation. Hence, thorough validation measures, clinician education, and institutional monitoring are vital for making sure that AI systems serve to support clinical judgment rather than replace it.

### 6.5 Remaining Gaps in Clinical Translation

Taken together, these hurdles portray a main difference between innovative methods and actual implementation in the clinic. Causal AI, in theory, has the potential to make a conceptual leap over traditional modeling, but still, the major part of the research in the area is limited to retrospective data sets, computer experiments, or conceptual originality. Access to casual AI in real‐world arrhythmia management via initiating prospective trials still remains a challenge. Consequently, the proof of the clinical effectiveness, the ability to generalize across health systems, as well as the long‐term patient outcomes are scarcely found. Besides, the legal requirement to have decision support systems driven by causal AI is still at the stage of development, and standardized validation schemes have not been generally accepted.

This calls for a combined effort in many areas if we were to think of solving these problems. Future inquiry should give primary importance to data‐driven validation, establishing uniform dataset protocols as well as developing collaborative data‐sharing frameworks that uphold patient confidentiality while at the same time allowing large‐scale causal analysis. On top of that, the interest of causal AI in clinical electrophysiology will only be supported by formulating clear regulatory and ethical codes of conduct. Presently, until we find solutions for these points of contention, it is better to consider causal AI a vehicle with highly likely features, but which has not been fully completed yet.

## 7 Future Directions and Opportunities

AI is rapidly transforming arrhythmia research by shifting from purely pattern‐recognition models toward frameworks that attempt to capture the underlying physiological mechanisms of cardiac rhythm disturbances. Across recent studies, several converging themes emerge: the integration of causal reasoning into deep learning models, the coupling of AI with mechanistic cardiac simulations, the expansion of real‐time monitoring through wearable technologies, and the development of collaborative learning frameworks using multimodal clinical data. While these approaches collectively suggest a path toward more interpretable and clinically actionable models, important methodological challenges remain regarding validation, integration across data modalities, and real‐world deployment.

### 7.1 Causal Deep Learning Approaches

Recently, the literature has leaned towards the fact that standard deep learning models, while very precise in classifying tasks, usually fall short in mechanistically interpreting the underlying processes. To try to overcome this shortcoming, a number of papers suggest the integration of causal inference or physiological modeling within the architectures of neural networks.

This is illustrated by Dalton et al. who showed that graph neural networks, when combined with neural ordinary differential equations, can describe cardiac motion and electrical activity as dynamic graph structures, which allows the reconstruction of spatiotemporal cardiac dynamics from imaging data [83]. In fact, this method shows how causal representations might go beyond just capturing surface‐level patterns in cardiac signals and instead reveal the latent physiological processes. In the same vein, Wang et al. developed Causal ECGNet, a model that lays out the causal relationships among ECG features such as RR intervals, QRS morphology, and heart rate variability. With these relationships integrated into convolutional and recurrent neural networks, the system was not only able to lessen the impact of confounders and raise the level of arrhythmia classification accuracy, but it also made the feature importance interpretations possible [72].

Such pieces of evidence harmonize with the very first piece from Hannun et al., which revealed the capability of deep neural networks to recognize complicated ECG patterns that are linked to arrhythmias, including minor changes in QRS morphology and irregularities in RR intervals [19]. On the other hand, this work also pointed out a major shortcoming of purely data‐driven approaches: while they can pick out predictive patterns, they are not designed to explain the physiological bases of those patterns. Therefore, a lot of the recent publications on this topic are focusing on causal modeling, which represents a path to reconcile predictive prowess with mechanistic interpretability. Still, methodological hurdles are present. At best, present causal deep learning models make the causal graph or physiological relationships a basis for assumptions, but these might fall short of describing the full intricacy of cardiac electrophysiology. Moreover, the majority of the models are developed based on limited or hand‐picked data sets, which makes it hard to predict these models' performance and reliability in other patient populations and clinical environments.

### 7.2 Integration With Mechanistic Cardiac Simulations

One of the new directions is the combination of ML and mechanistic cardiac simulations. In fact, mechanistic models provide a detailed depiction of electrophysiological phenomena, like conduction velocity, tissue heterogeneity, and distribution of fibrosis, which are the main factors in the initiation and continuation of arrhythmia. In this respect, Colebank et al. stated that mechanistic modeling offers a physiologically based approach to understanding arrhythmia mechanisms and to conducting virtual testing on patient‐specific cardiac structures [84].

In fact, by merging clinical data, such as ECG recordings and structural imaging, with computational models, scientists are able to predict how changes in tissue properties or conduction pathways may lead to the occurrence of arrhythmia.

Scientists are increasingly of the view that coupling mechanistic models and AI is going to be better for both interpretability and prediction. While ML models can handle and analyze big data sets very quickly, mechanistic simulations might be able to explain the cause of the predictions. Despite this, the actual combination is far from simple. Mechanistic simulations require a lot of computational power and usually need patient‐specific anatomical data, which is often not available in normal clinical cases. As a result, designing hybrid models that can find an optimal balance between computational efficiency and physiological accuracy is going to be an important area of focus in research.

### 7.3 Real‐Time Monitoring and Wearable Technologies

One more significant area where causal AI could be utilized is the proliferation of wearable cardiac monitoring devices. Physiological monitoring done continuously can record even the very short or occasional arrhythmias which may not be detected during the standard clinical tests.

Several large‐scale studies have established that wearable‐based arrhythmia detection is indeed doable. Perez et al. demonstrated that a smartwatch can, with a very high positive predictive value, discover AF by measuring the pulse continuously in the population of users on an extremely large scale [85]. Another, more meaningful, work by Zertal et al. proposed a deep learning solution working in real‐time and capable of changing with the ECG signals over time. It also provides dynamic risk estimation and a preemptive recognition of cardiovascular events [86]. Reviews of wearable ECG technologies point out their increasing diagnostic capacity of especially in long‐term monitoring and early detection of sporadic arrhythmias [87]. All of these research results refute the wearable devices being simply another tool for catching arrhythmias, but rather believe that these could be great components of future surveillance systems.

Still, the wearables are not perfect yet, and their limited use has been justified with the following reasons. First, wearables are very sensitive to the user's movements, which leads to the signal being vulnerable to artifacts that even the best motion sensors cannot completely eliminate. Secondly, wearable data collects very large data sets which pose challenges related to data processing, storage, and privacy. However, the combination of wearable technologies with causal inference can play a pivotal role in solving the above‐mentioned problems and so drastically change the diagnostic field. An important corollary is that real‐world actuation and confirmatory analysis are highly warranted for a full exploration of these benefits.

### 7.4 Multimodal and Federated Causal Learning

Additionally, where there is a growing agreement is that it's very important to bring together many patient data sources. The risk of heart rhythm disorder is affected by an intricate mix of electrical signals, the heart's structural features, clinical history, and lifestyle factors. Multimodal AI models try to understand these aspects by merging different data types into a single analytical framework. Alasmari et al. have come up with a federated learning architecture that combining ECG signals, cardiac imaging, electronic health records, and lifestyle variables to enhance early detection and personalized management of cardiac disease [88]. In this setup, causal inference methods figure out how different risk factors lead to arrhythmia outcomes while keeping patient privacy by decentralizing model training.

In the same way, Goto et al. have shown that multinational federated learning networks are feasible for training ECG and echocardiography models across multiple institutions [89]. These kinds of collaborative techniques allow the use of huge and diverse data sets without the direct sharing of sensitive patient information. These methods could be a solution to a long‐standing problem of clinical AI models, where they often show a drop in performance when used in populations that are different from those they were trained on. Nonetheless, there are still major issues that need to be overcome. Federated learning systems should be able to handle data quality heterogeneity, differences in clinical protocols across institutions, and variations in patient demographics. Also, it is very complicated to unify causal frameworks over multiple data sets, and this certainly calls for further methodological work.

### 7.5 Translational and Clinical Integration

Ultimately, the real benefit of causal AI methods is their capacity to facilitate clinical decision‐making. Usually, traditional predictive models are like “black boxes” that can create misunderstanding among clinicians and might even prevent their usage in healthcare systems. Causal inference models can be an answer to this problem because they emit explanations that are physiologically comprehensible. Gaies et al. showed how causal inference approaches enable clinical decision support systems to comprehend patient‐specific risk factors for arrhythmia development [90]. The system used variable interactions, such as electrolyte abnormalities, comorbidities, and QT interval measurements, together with the assertion that arrhythmia risk is driven by these factors to produce interpretable explanations for elevated arrhythmia risk, and achieved higher early detection rates with respect to standard monitoring strategies. While these findings indicate a possible clinical translation, broad‐scale deployment of clinical decision support tools remains a difficult task with several challenges still to be addressed. For example, clinical decision support tools must be subjected to comprehensive validation using patient populations of diverse CAS and integrate very well with electronic health record systems, besides meeting regulatory requirements of medical software. Additionally, for model output to be trusted and interpreted by clinicians requires that the model design is made transparent and causal relationships are communicated clearly (Table 3).

**Table 3:** Future directions in causal AI for arrhythmia.

Direction	Approach	Expected benefit	References
Causal deep learning	Integrating causal graphs into CNNs, GNNs	Improves interpretability and accuracy	[83, 84]
Mechanistic integration	Combining AI with cardiac electrophysiology simulations	Enables patient‐specific predictions	[85]
Wearable causal inference	Real‐time causal modeling from continuous ECG	Enables proactive arrhythmia prevention	[86, 87, 88]
Federated Causal learning	Privacy‐preserving, cross‐institutional model training	Enhances generalizability and inclusivity	[89, 90]
Clinical translation	Decision‐support with causal explainability	Builds clinician trust and usability	[90]

### 7.6 Remaining Challenges and Research Gaps

Several major concerns appearing through all these fields have been identified in general. Firstly, numerous causal AI frameworks are being tested mostly on historical data sets, which might not reflect clinical workflows. Hence, prospective clinical trials are required to demonstrate clinical reliability. Secondly, combining diverse data types such as ECG signals, imaging, and electronic health records remains a very difficult task. It will be necessary to standardize data representations and to have interoperable frameworks so that we can take full advantage of multimodal causal learning. Finally, even though causal AI systems are expected to be more understandable, it is still quite a challenge to correctly define causal links in such a complex biological system as the human body. Cardiac electrophysiology is affected by complicated interactions between the cellular, structural, and systemic levels, and currently, the models do not reveal accurately these aspects.

Progressing in these areas will take teamwork between healthcare providers, computational scientists, and biomedical engineers. When done well, causal AI methods may bring arrhythmia studies from just forecasting to gaining insights through mechanisms, which will lead to more accurate diagnostics, treatment plans tailored to the individual, and better healthcare results.

## 8 Conclusion

Causal AI signifies a hopeful transformation in cardiac arrhythmia studies. This approach changes the focus from merely predicting based on correlations to actually uncovering the physiological mechanisms causing arrhythmias. Fusing causal inference with electrophysiology, imaging, and clinical data is the way through which the field can identify real disease drivers, foresee treatment responses, and ultimately come up with patient‐centric management plans. Conceptually and practically, the use of causal ECG modeling, virtual cardiac twins, and counterfactual treatment simulations serves as an example of how computer‐based models can convert multifaceted heart health data into understandable clinical pieces that facilitate diagnosis, risk assessment, and selection of therapy.

However, widespread clinical use of causal AI is not yet a reality because of the challenges ahead. One key area for research is the compilation of extensive longitudinal data sets, which record interventions in detail along with outcomes, thereby allowing for better causal analyses. Besides that, it is crucial to integrate data from various modalities, carry out clinical trials that validate the use of causal AI, and create models that, besides being causal, are also humanly understandable in order to gain clinicians' confidence and show the benefits in real life. Last but not least, laws and ethical principles will have to be reconsidered to make sure the deployment of causal AI is safe, just, and responsible. By means of teamwork among clinicians, computational scientists, and authorities, causal AI is capable of making arrhythmia research more aligned with mechanistic understanding and personalization of cardiovascular care.

## Funding

The authors have nothing to report.

## Ethics Statement

The authors have nothing to report.

## Consent

The authors have nothing to report.

## Conflicts of Interest

The authors declare no conflicts of Interest.

## Data Availability

The authors have nothing to report.
